# Fruit, vegetable, and fruit juice consumption and risk of gestational diabetes mellitus: a systematic review and meta-analysis: List of all authors

**DOI:** 10.1186/s12937-023-00855-8

**Published:** 2023-05-20

**Authors:** Yan-Ping Liao, Qing-Xiang Zheng, Xiu-Min Jiang, Xiao-Qian Chen, Xiao-Xia Gao, Yu-Qing Pan

**Affiliations:** 1 School of Nursing, Fujian Medical University, No. 1 Xuefu North Road, University Town, Shangjie Zhen, Minhou County, Fuzhou City, 350000 Fujian Province China; 2 Fujian Maternity and Child Health Hospital College of Clinical Medicine for Obstetrics & Gynecology and Pediatrics, Fujian Medical University, No. 18 Daoshan Street, Gulou District, Fuzhou City, 350000 Fujian Province China; 3 Fujian Obstetrics and Gynecology Hospital, Zhanban Street, Jinan District, Fuzhou City, 350000 Fujian Province China

**Keywords:** Fruit, Vegetable, Fruit juice, GDM, Gestational diabetes mellitus

## Abstract

**Background:**

Fruit, vegetable, and fruit juice intake is associated with the risk of gestational diabetes mellitus (GDM). However, the conclusion is limited and conflicted. The purpose of this systematic review and meta-analysis is to investigate the association between fruit, vegetable, and fruit juice consumption and the risk of GDM.

**Methods:**

To find relevant studies, we searched PubMed, The Cochrane Library, Web of Science, Embase, ScienceDirect, PsycINFO, CINAHL, Ovid, EBSCO, CBM, CNKI, Wanfang Data, and VIP for the report on prospective cohort studies published from inception to April 8, 2022. Summary relative risks (*RR*) and 95% confidence intervals (*Cis)* were estimated using a random-effects model.

**Results:**

A total of 12 studies with 32,794 participants were included in the meta-analysis. Total fruit consumption was associated with a lower risk of GDM (*RR* = 0.92, 95% *CI* = 0.86–0.99). Whereas an increasing the consumption of vegetable, including all vegetable (*RR* = 0.95, 95% *CI* = 0.87–1.03), starchy vegetable (*RR* = 1.01, 95% *CI* = 0.82–1.26), and fruit juice (*RR* = 0.97, 95% *CI* = 0.91–1.04) was not associated with a reduction in the risk of GDM. In a dose‒response analysis of eight studies, a 3% reduction in risk of GDM for a 100 g/d increase in fruit consumption (*RR* = 0.97, 95% *CI* = 0.96–0.99).

**Conclusions:**

The findings suggest that higher fruit consumption may reduce the risk of GDM, with a 3% reduction in the risk of GDM for every 100 g/d increase in fruit intake. Higher-quality prospective studies or randomized clinical trials are required to validate the effect of different variations of fruits, vegetables, and fruit juice consumption on the risk of GDM.

**Supplementary Information:**

The online version contains supplementary material available at 10.1186/s12937-023-00855-8.

## Introduction

Gestational diabetes mellitus (GDM) is an endocrine disorder in which abnormal blood glucose first occurs or is detected during pregnancy [1]. The International Diabetes Federation (IDF) estimates that 16.7% of women aged 10–49 years currently have GDM [2]. GDM is also linked to an increased risk of both short and long-term adverse pregnancy outcomes, including large birth weight, baby-obstructed labour, and type 2 diabetes mellitus (T2DM) in both mother and offspring [3]. The increased prevalence and adverse outcomes of GDM have caused a serious societal, economic, and health burden on both the population and individuals.

There is growing interest in the function of dietary behaviour and patterns in the development of chronic diseases [4, 5]. Fruit and vegetable consumption have been linked with a reduced incidence of cardiovascular diseases, metabolic syndrome, and type 2 diabetes [6, 7]. Fruit juices have become part of the daily diet and this is another important form of fruit intake. Many people try to supplement their daily fruit intake by drinking fruit juice. Drinking fruit juice may be an easy and effective way to reach your goal of 5 servings of fruit per day [8]. In another study, pregnant women reported a preference for drinking homemade fruit juice, and they believed the juice had more nutrients, such as vitamins and minerals, compared to whole fruit [9].The intake of fruits, vegetables, and fruit juices could explain some of the beneficial effects of the individual components and nutrients in the daily diet [10, 11]. Fruits and vegetables have anti-inflammatory properties and are rich in antioxidants, dietary fibre, and healthful phytochemicals [12, 13]. These compounds could improve insulin sensitivity by reducing pancreatic β cell apoptosis, muscular inflammation, and oxidative stress [14, 15].

The World Health Organization (WHO) suggests that consuming more than 400 g of different fruits and vegetables per day may reduce the incidence of diabetes [16]. Consumption of different types and amounts of fruits and vegetables could provide different levels of diabetes protection [17]. Evidence indicates that the Mediterranean diet (i.e.,vegetables and fruit-rich foods) could significantly reduce the development of GDM [18, 19]. Compared with a red-meat diet, vegetable and fruit-rich dietary patterns were linked to a reduced occurrence of impaired fasting glucose [20]. Consumption of fruit or green leafy vegetables is related to reduced risk of GDM [21, 22]. However, when compared to other cruciferous vegetables, legumes, or whole-grain foods, higher levels of potato consumption before pregnancy were found to be associated with a higher risk of GDM [23]. There is only one systematic review that indicated that fruit intake before pregnancy is associated with the risk of GDM, but this review did not assess the effects of vegetable or fruit juices on the incidence of GDM [24]. Therefore, based on the inconsistency among studies and to solve the limitations of the current review, we conducted a systematic review and meta-analysis to assess the effect of fruit, vegetable, and fruit juice intake on the risk of GDM and to assist in the exploration of dietary intervention strategies.

## Methods

### Search strategy

We conducted and reported this systematic review according to the guidelines of the Preferred Reporting Items for Systematic Reviews and Meta-analysis (PRISMA) 2020 statement (see online supplementary materials, Table S1). A comprehensive literature search was conducted in the following databases: PubMed, The Cochrane Library, Web of Science, Embase, Scopus, Ovid, EBSCO, Wanfang Data, CNKI, and VIP. There were no specified language restrictions. The database search was conducted from the database inception dates to April 8, 2022. During the retrieval process, MeSH terms were used as follows: “pregnancy or pregnant* or gestation*” and “vegetables or vegetable or fruits or fruit or vegetable juices or juice, vegetable or juices, vegetable or vegetable juice or fruit juices or juices, fruit”, and “diabetes, gestational or diabetes, pregnancy-induced or diabetes, pregnancy-induced or pregnancy-induced diabetes or gestational diabetes or diabetes mellitus, gestational or gestational diabetes mellitus” (see online supplementary materials, Table S2). References of original publications as well as previous meta-analyses or reviews were also manually reviewed.

### Study selection and inclusion and exclusion criteria

Duplicate articles were removed in EndNoteX9. Two reviewers (Liao and Zheng) screened titles and abstracts to determine eligibility. Disagreements were adjudicated by a third reviewer (Jiang). Based on the title and abstract screening, the full-text articles of all the eligible studies were reviewed by Liao and Zheng.

### Inclusion and exclusion criteria

The criteria of the included and excluded studies were guided by the PECOs. The following criteria were used to determine which studies to include in the meta-analysis. (1) Participants: eligibility criteria were restricted to pregnant adult women (18 years old and above); (2) Exposures: the intake of fruit, vegetables, and fruit juice was the exposure; (3) Outcomes: the GDM criteria included the following two methods: incident cases defined by self-reported clinical diagnosis of GDM or by meeting the criteria of either a fasting blood glucose concentration of 92 mg/dL, a 1-hour blood glucose concentration of 180 mg/dL, or a 2-hour blood glucose concentration of 153 mg/dL after a glucose tolerance test. (4) Study design: cohort studies. Studies that do not report relative risk and 95% confidence intervals for the relationship between fruit, vegetable, or/and fruit juice consumption and GDM risk were excluded.

### Data extraction

The researchers extracted authors, year of publication, country/location, follow-up period, number of participants, number of GDM cases, age, pre-BMI, exposure (fruit/all vegetables/ starchy vegetable/fruit juice), assessment of GDM, exposure assessment, quality assessment score, and adjustments. The information was extracted by Liao and Zheng and any disagreements were solved through discussion with Jiang. When studies reported multivariate models, we included the highest exposure of the adjusted variables in the risk estimates.

### Quality assessment

Evaluation of cohort studies was performed using the Newcastle‒Ottawa Scale (NOS) [25]. There are three categories and a maximum of nine points: (1) selection of populations; (2) comparability of the two groups; and (3) assessment of outcome. Studies with different points were divided into high quality (7–9 points), moderate quality (5–6 points), and poor quality (0–4 points). The quality of each study was assessed by Liao and Zheng, and any inconsistencies were discussed with Jiang. Studies was not excluded from the meta-analysis based on quality assessment scores.

### Statistical analysis

STATA software version 16 was used for statistical analyses. For the meta-analysis, we used the study-specific relative risk (*RR*) and 95% confidence interval (*Cis*) for the highest versus lowest category of fruit, vegetables, and fruit juice intake with a random-effects model analysis. Statistical significance was defined as a two-tailed *P* value less than 0.05. The *I*^*2*^ (*P* < 0.1) and *Q* statistics were used to assess heterogeneity between studies. *I*^*2*^ values of 25%, 50%, and 75% represent low, moderate, and high heterogeneity, respectively. To investigate sources of heterogeneity, we conducted subgroup analyses by the period of dietary assessment (prepregnancy/first trimester/second trimester), location (Asia/Non-Asia), total number of participants (≥ 2000/<2000), number of GDM (≥ 500/<500), pre-BMI (< 25/≥25 kg/m^2^), parity-adjusted (yes/no), family history of diabetes-adjusted (yes/no), physical activity-adjusted (yes/no), smoking-adjusted (yes/no), alcohol-adjusted (yes/no). We assessed the stability of the study results by the trim and fill method in a sensitivity analysis or by excluding studies at a high risk of bias. Begg’s test and Egger’s test or funnel plot asymmetry were used to examine publication bias.

In the dose‒response analysis, we used generalized least squares to calculate study-specific slopes and 95% confidence intervals [26]. Statistical significance was defined as a *P* value of 0.05. For each category, the mean or median fruit, vegetable, and fruit juice intake were assigned to the appropriate *RR* for the individual study. We used the midpoint between the highest and lowest bounds in each category when the data on average consumption were not available. In an open category, if only the value of the highest category or the lowest category is known, we assume that the lowest bound is zero and the highest category bound is 1.5 times the lowest category [27, 28]. When the study used the number of servings to express the intake of vegetables and fruits, we normalized it to one serving equal to106 grams [29].

**Fig. 1:**
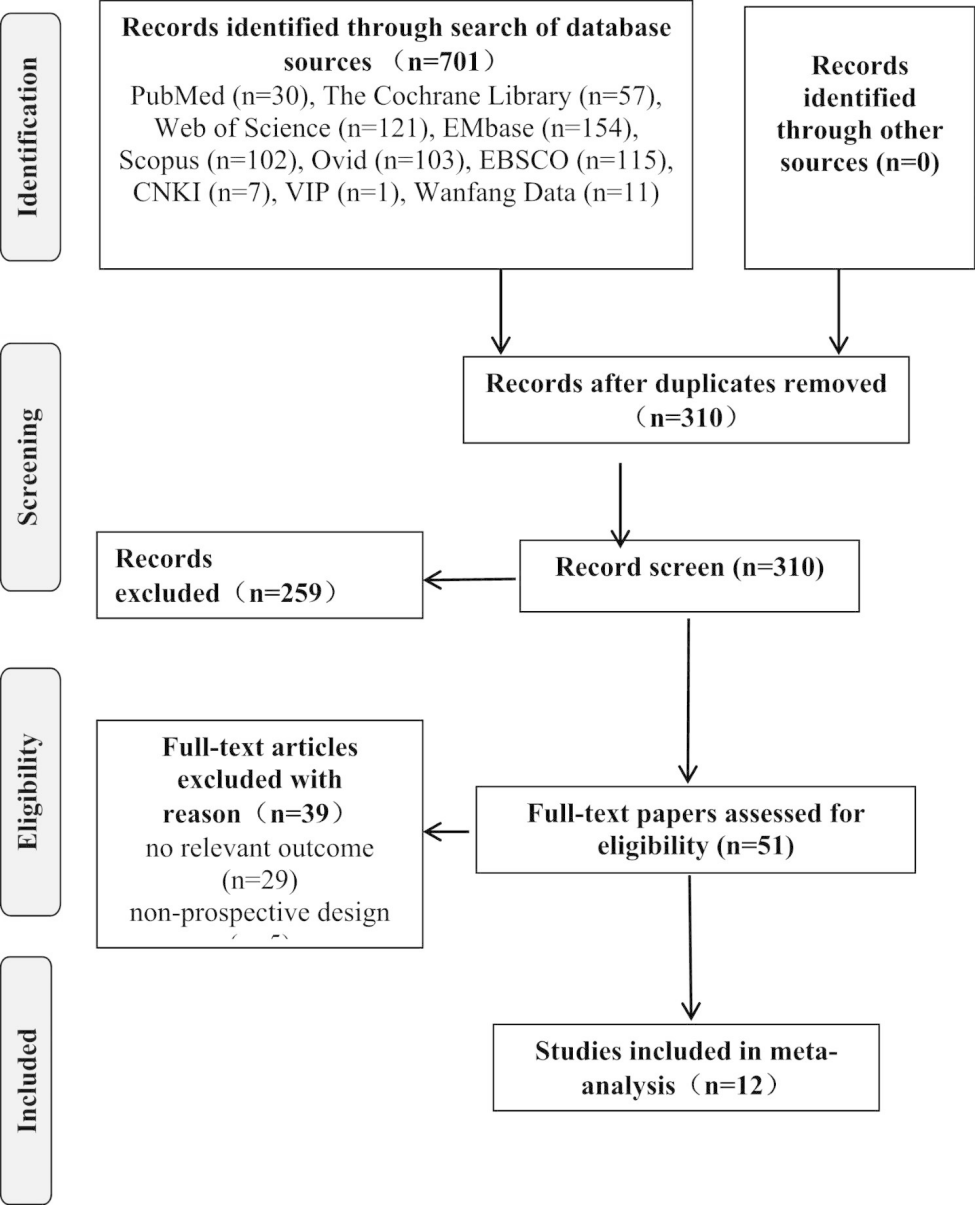
PRISMA flow chart of study selection

**Fig. 2:**
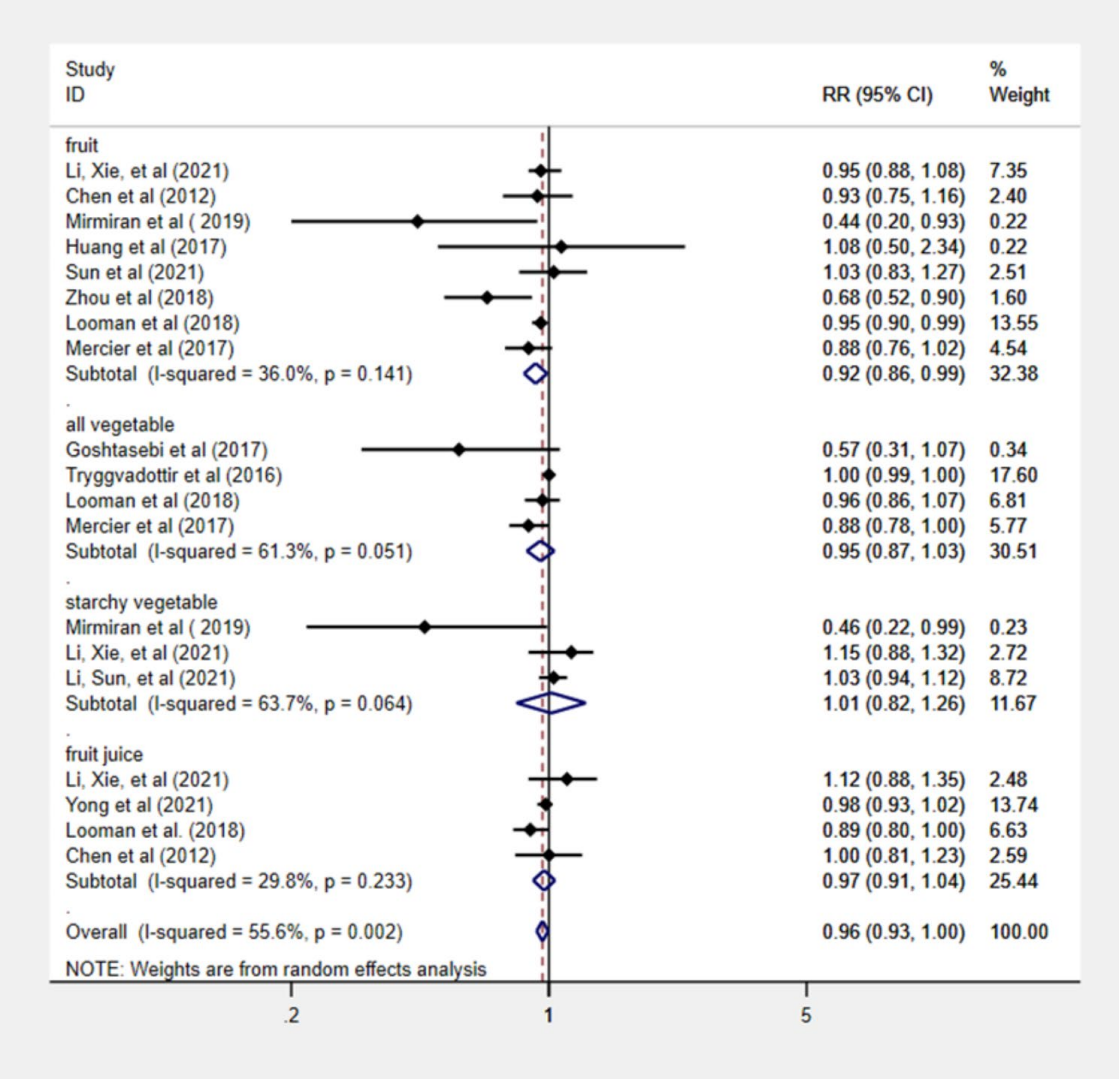
Forest plot of fruit, vegetable, fruit juice intake, and risk of GDM.

**Table 1:** Characteristics of the included studies

Author/publication year	Country/Location	Follow-up period^1^	Total number	Number of GDM	Age (year)	Pre-BMI (kg/m^2^)	Exposure	Assessment of GDM	Exposure assessment	Quality	Adjustments
Li, Xie, et al. 2021	China	1	2,987	405	28.5 ± 3.6	21.3 ± 1.3	Fruits, starchy vegetables, Fruit juice	fasting glucose concentration	food frequency questionnaire	6	adjusted for age, pre-pregnancy BMI, family history of diabetes, physical activity, fiber intake, and meat intake.
Chen et al. 2012	America	12	13,475	860	24–44	unspecified	Fruits, Fruit juice	self-reported diagnosis of GDM	semi-quantitative food frequency questionnaire	6	adjusted for age, race/ethnicity, pre-pregnancy BMI, parity, family history of diabetes, physical activity, alcohol intake, smoking, intake of cereal fiber, processed meat, red meat, sugar-sweetened beverages, and fruit juice.
Mirmiran et al. 2019	Iran	1.3	1,026	71	26.7 ± 4.3	25.4 ± 4.5	Fruits, All vegetables	100 g 3-hour oral glucose tolerance test	semi-quantitative food frequency questionnaire	6	adjusted for age, level of education, pre-pregnancy BMI, third gestational weight gain, history of GDM, family history of GDM, total energy intake, total fat, total fiber, magnesium, cholesterol intake
Huang et al. 2017	China	1	772	169	26.0 ± 3.2	19.7 ± 2.5	Fruits	75 g 2-hour oral glucose tolerance test	3-day food record	6	adjusted for age, level of education, occupation, pre-pregnancy BMI, gestational weight gain, family history of diabetes, income level, smoking status and alcohol, energy intake, and the consumption of grain, vegetables, meat, and fish.
Sun, et al. 2021	China	1	1,453	523	28.5 ± 4.0	20.7 ± 2.7	Fruits	75 g 2-hour oral glucose tolerance test	3-day food record	6	adjusted for age, level of education, family income level, pre-pregnancy BMI, parity, family history of diabetes, physical activity, smoking, alcohol drinking, whole energy, vegetables, grains, beverage, dietary fiber, and different subtypes of fruit intake.
Zhou et al. 2019	China	3	3,330	370	28.2 ± 3.4	20.7 ± 2.7	Fruits	75 g 2-hour oral glucose tolerance test	food frequency questionnaire	6	adjusted for age, ethnology, average personal income, level of education, pre-pregnancy BMI, gestational weight gain before GDM diagnosis, parity, family history of diabetes, family history of obesity, physical activity, smoking, alcohol, total energy intake, vegetables, whole grains, red meat, fish, eggs, dairy products.
Looman et al. 2018	Australian	5	6,263	285	27.5 ± 1.5	23.9 ± 4.6	Fruits, All vegetables, Fruit juice	50 g 2-hour oral glucose tolerance test	food frequency questionnaire	7	adjusted for age, level of education, pre-pregnancy BMI, parity, polycystic ovarian syndrome, hypertension during pregnancy, physical activity, smoking, and total energy intake.
Mercier et al. 2019	Canada	10	398	126	36.6 ± 5.1	27.9 ± 6.8	Fruits, All vegetables	75 g 2-hour oral glucose tolerance test	food frequency questionnaire	7	adjusted for age and pre-pregnancy BMI.
Yong et al. 2021	Malaysia	2	452	48	30.0 ± 4.4	23.7 ± 4.8	Fruit juice,	75 g 2-hour oral glucose tolerance test	semi-quantitative food frequency questionnaire	7	adjusted for age, pre-pregnancy BMI, parity, and gestational weight gain, total energy.
Goshtasebi et al. 2018	Iran	1	1,026	708	26.7 ± 4.3	25.4 ± 4.5	Starchy vegetables	100 g 3-h oral glucose tolerance test	semi-quantitative food frequency questionnaire	6	adjusted for age, level of education, pre-pregnancy BMI, history of GDM, family history of diabetes, total energy intake, total fiber, and cholesterol intake.
Li, Sun, et al. 2021	China	1	1,444	520	28.6 ± 4.0	20.6 ± 3.0	Starchy vegetables	75 g 2-hour oral glucose tolerance test	3-day food record	6	adjusted for age, level of education, pre-pregnancy BMI, parity, family history of diabetes, physical activity, smoking, alcohol consumption, total energy intake, and the consumption of whole grains, fruits, meat, fish, and other vegetables.
Tryggvadottir et al. 2016	Iceland	1.5	168	17	29.0 ± 4.8	28.6 ± 1.8	All vegetables	75 g 2-hour oral glucose tolerance test	4-day weighed food record	6	adjusted for age, pre-pregnancy BMI, parity, energy intake, weekly weight gain, and total met.

Note 1: the followed-up period of this study included the recruitment time and the observation time of the outcome indicators

All vegetables: all types of vegetables consumed in the daily diet, including starchy vegetables and other vegetables. Such as tomato, tomato sauce, capsicum (bell or sweet peppers), lettuce, cucumber, celery, beetroot, carrots, cabbage, cauliflower, broccoli, spinach, peas, green beans, bean sprouts, pumpkin, onion, garlic, mushrooms, zucchini, potato, etc.

Starchy vegetables: include potatoes, pumpkin, lotus root, yam, taro, water chestnut, pea, and cowpea, etc.

**Table 2:** Meta-analysis of intake of fruit, vegetables, and fruit juice and risk of GDM

Variables	Number of participants	Cases/total	Test of association		Test of heterogeneity		Analysis of publication bias	
			*RR* (95% *CI*)	*P* value	Heterogeneity (*I*^*2*^, %)	*P* value	Begg’s test (*P* value)	Egger’s test (*P* value)
Fruit	29,704	2809	0.92 (0.86, 0.99)	0.02	36.0%	0.14	0.22	0.19
All vegetable	5457	1633	0.95 (0.87, 1.03)	0.24	61.3%	0.23	0.17	0.06
Starchy vegetable	7855	499	1.01 (0.82, 1.26)	0.90	63.7%	0.90	0.60	0.62
Fruit juice	23,177	1598	0.97 (0.91, 1.04)	0.37	29.8%	0.23	0.50	0.87

All vegetables: including starchy vegetables and other vegetables

**Table 3:** Subgroup analyses for the association between vegetable, fruit, and fruit juice consumption and the risk of GDM

Variables/ Subgroup	Fruit			All vegetable				Starchy vegetable				Fruit juice				
	Number of studies	*RR* (95% *CI*)	*p*	*I* ^*2*^	Number of studies	*RR* (95% *CI*)	*p*	*I* ^*2*^	Number of studies	*RR* (95% *CI*)	*p*	*I* ^*2*^	Number of studies	*RR* (95% *CI*)	*p*	*I* ^*2*^
Period of dietary assessment																
Pre-pregnancy	3	0.91 (0.77, 1.08)	0.28	48.3%	2	0.81 (0.50, 1.31)	0.39	62.1%	1	0.46 (0.22, 0.98)	0.04	---	2	0.91 (0.83, 1.01)	0.07	0%
First trimester	2	0.93 (0.80, 1.08)	0.36	29.7%	1	0.88 (0.78, 1.00)	0.04	---	1	1.03 (0.94, 1.12)	0.51	---	---	---	---	---
Second trimester	3	0.85 (0.65, 1.11)	0.24	61.5%	1	1.00 (0.99, 1.01)	1.00	---	1	1.15 (0.94, 1.14)	0.18	---	2	1.00 (0.91, 1.11)	0.93	30.0%
Location																
Asia	5	0.87 (0.71, 1.06)	0.15	59.3%	1	0.57 (0.31, 1.06)	0.07	---	2	1.05 (0.97, 1.14)	0.25	0%	2	1.00 (0.91, 1.11)	0.93	30.0%
Non-Asia	3	0.94 (0.90, 0.99)	0.00	0%	2	0.96 (0.90, 1.04)	0.32	56.4%	1	0.46 (0.22, 0.98)	0.04	---	2	0.91(0.83, 1.01)	0.07	29.8%
Participants																
≥ 2000	4	0.92 (0.85, 1.00)	0.05	46.3%	1	0.96 (0.86, 1.07)	0.46	---	1	1.15 (0.94, 1.41)	0.18	---	3	0.97 (0.85, 1.12)	0.71	47.5%
< 2000	4	0.91 (0.74, 1.11)	0.34	41.5%	3	0.92 (0.80, 1.07)	0.29	72.3%	2	0.75 (0.35, 1.63)	0.47	77.0%	1	0.98 (0.94, 1.03)	0.39	0%
Cases																
≥ 500	2	0.98 (0.84, 1.14)	0.79	0%	1	0.57 (0.31, 1.06)	0.08	---	1	1.03 (0.94, 1.12)	0.51	---	1	1.00 (0.81, 1.23)	1.00	0%
< 500	6	0.90 (0.82, 0.99)	0.02	50.9%	3	0.96 (0.90, 1.04)	0.32	56.4%	2	0.78 (0.32. 1.90)	0.59	81.2%	3	0.97 (0.88, 1.06)	0.47	52.4%
Pre-BMI																
< 25	5	0.94 (0.86, 1.01)	0.10	36.8%	3	0.96 (0.86, 1.07)	0.46	---	2	1.05 (0.97, 1.14)	0.25	0%	3	0.97 (0.88, 1.06)	0.47	52.4%
≥ 25	2	0.69 (0.36, 1.32)	0.26	66.8%	1	0.92 (0.80, 1.07)	0.29	72.3%	1	0.46 (0.22, 0.98)	0.04	---	---	---	--	---
Parity-adjusted																
Yes	7	0.92 (0.85, 0.99)	0.02	44.5%	3	0.96 (0.90, 1.04)	0.32	56.4%	3	1.01 (0.82, 1.26)	0.90	63.7%	4	0.97 (0.91, 1.04)	0.37	29.8%
No	1	1.08 (0.50, 2.34)	0.84	0%	1	0.57 (0.31, 1.06)	0.08	---	---	---	---	---	0	---	---	---
Family history of diabetes-adjusted																
Yes	5	0.92 (0.81, 1.04)	0.17	35.5%	1	0.57 (0.31, 1.06)	0.08	---	3	1.01 (0.82, 1.26)	0.90	63.7%	2	1.06 (0.91, 1.23)	0.46	0%
No	3	0.90 (0.78, 1.04)	0.14	57.5%	3	0.96 (0.90, 1.04)	0.32	56.4%	---	---	---	---	2	0.95 (0.87, 1.04)	0.23	59.1%
Physical activity-adjusted																
Yes	5	0.94 (0.87, 1.01)	0.07	35.9%	1	0.96 (0.86, 1.07)	0.46	---	2	1.05 (0.97, 1.14)	0.25	0%	3	0.97 (0.85, 1.12)	0.71	47.5%
No	3	0.80 (0.55, 1.18)	0.27	49.2%	3	0.92 (0.80, 1.07)	0.29	72.3%	1	0.46 (0.22, 0.98)	0.04	---	1	0.98 (0.91, 1.04)	0.39	0%
Smoking-adjusted																
Yes	5	0.92 (0.83, 1.03)	0.16	36.9%	1	0.96 (0.86, 1.07)	0.46	---	1	1.03 (0.94, 1.12)	0.51	---	2	0.91 (0.83, 1.01)	0.07	0.0%
No	3	0.89 (0.76,1.04)	0.15	53.1%	3	0.92 (0.80, 1.07)	0.29	72.3%	2	0.78 (0.32, 1.90)	0.59	81.2%	2	1.00 (0.91, 1.11)	0.93	30.0%
Alcohol-adjusted																
Yes	4	0.89 (0.73,1.09)	0.27	48.8%	0	---	---	---	1	1.03 (0.94, 1.12)	0.51	---	1	1.00 (0.81, 1.23)	1.00	0%
No	4	0.93 (0.87,1.00)	0.05	36.7%	4	0.95 (0.87, 1.03)	0.24	61.3%	2	0.78 (0.32, 1.90)	0.59	81.2%	3	0.97 (0.88, 1.06)	0.47	52.5%

All vegetables: including starchy vegetables and other vegetables

**Fig. 3:**
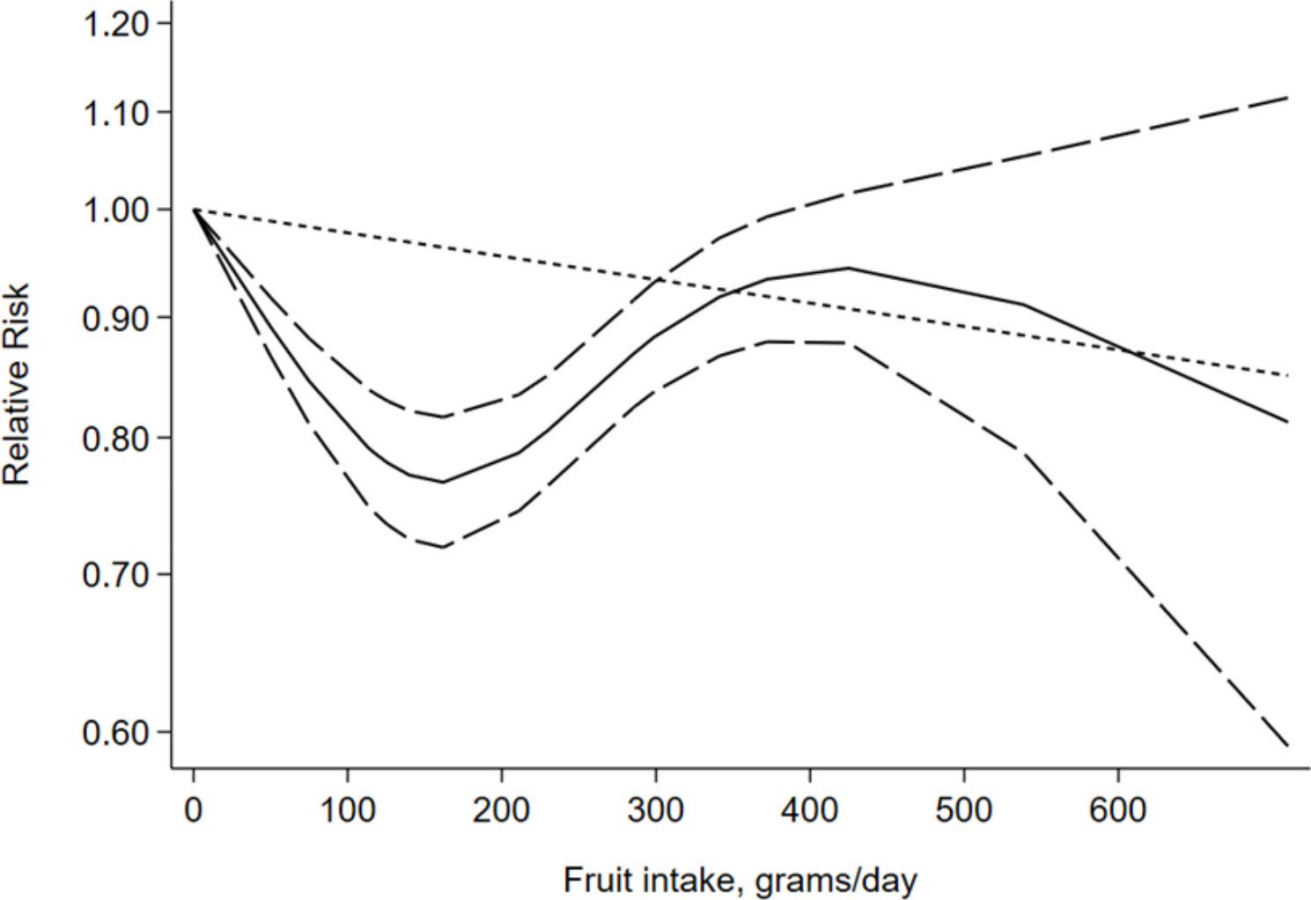
Dose-response analyses of fruit intake and risk of GDM.

## Results

### Study characteristics

The study selection process is shown in Fig. 1. In total, 701 articles were retrieved from 10 database searches. The eligibility of 320 articles was determined after removing duplicate publications. There were 51 studies eligible for full-text review, and 12 studies met the inclusion criteria. The study characteristics are summarized in Table 1. The studies were all cohort studies. The 12 studies were published between 2012 and 2021 and were conducted in eight countries, one was conducted in America [30], one in Australia [31], five in China [9, 32–35], one in Canada [36], two in Iran [37, 38], one in Iceland [39], and one in Malaysia [40]. Recruiting or follow-up periods ranged from one to 12 years. Two of these studies were from large cohort studies with longer recruitment times, resulting in the longer overall follow-up of the studies, and their study data results are only a portion of the large cohort studies [30, 36]. The period of dietary investigation for all the studies included three periods, four studies before pregnancy [30, 31, 37, 38], three studies in the first trimester [34–36], and five studies in the second trimester [9, 32, 33, 39, 40]. Eight studies provided information on fruit intake [9, 30–33, 35, 36, 38]. A total of seven studies reported on vegetable intake, of which four reported on all vegetables (including starchy vegetables and other vegetables) [31, 36, 38, 39], and three only reported on starchy vegetables [9, 34, 37]. Four studies reported on fruit juice intake [9, 30, 31, 40]. For the quality assessment, nine studies had a score of 6, indicating moderate quality [9, 30, 32–35, 37–39], and three studies had a score of 7, indicating high quality [31, 36, 40].

### Fruit consumption and risk of GDM

A total of eight studies involving 2,809 GDM outcomes and 28,604 participants reported an association between fruit consumption and GDM [9, 30–33, 35, 36, 38]. Summarizing all eight comparisons with a random effects model, fruit intake was inversely associated with the risk of GDM (*RR* = 0.92; 95% *CI*: 0.86–0.99). Participants in the highest intake quartile had an 8% lower risk of developing GDM than those in the lowest intake quartile. There was low heterogeneity among studies (*P* = 0.14, *I*^*2*^ = 36.0%) (Fig. 2; Table 2). Fruit intake increased by 100 g per day was linked to a 3% lower risk of GDM in a dose‒response meta-analysis (*RR* = 0.97, 95% *CI*: 0.96–0.99) (Fig. 3).

### Vegetable consumption and risk of GDM

A total of seven studies involving 2,132 GDM outcomes and 13,212 participants reported a link between vegetable consumption and GDM risk [9, 31, 34, 36–39]. The results indicated that there was no relationship between the intake of various types of vegetables and the risk of developing GDM. Four of these studies investigated the intake of all vegetables (including starchy vegetables and other vegetables) (*RR* = 0.95; 95% *CI*: 0.87–1.03) [31, 36, 38, 39] and three studies determined the intake of starchy vegetables (*RR* = 1.01; 95% *CI*: 0.82–1.26) [9, 34, 37] (Fig. 2; Table 2). Studies showed significant heterogeneity (all vegetables: *P* = 0.23, *I*^*2*^ = 61.3%; starchy vegetables: *P* = 0.90, *I*^*2*^ = 63.7%) (Fig. 2; Table 2). No correlation between the incidence of GDM and a 100 g/day increase in all vegetable intake (*RR* = 1.00, 95% *CI*: 0.99-1.00) and starchy vegetable intake (*RR* = 0.97, 95% *CI*: 0.93–1.01) was detected in dose‒response study (see online supplementary materials, Figure S1, S2).

### Fruit juice consumption and risk of GDM

In four studies, 1,598 people with GDM outcomes and 23,177 participants showed an association between fruit juice intake and the risk of GDM [9, 30, 31, 40]. No relationship was identified between fruit juice intake and the risk of developing GDM (*RR* = 0.97; 95% *CI*: 0.91–1.04). Low heterogeneity was detected among studies (*P* = 0.23, *I*^*2*^ = 29.8%) (Fig. 2; Table 2). There was no linear relationship between each 100 ml/day increase in fruit juice consumption and the risk of GDM based on a dose‒response analysis (*RR* = 1.01, 95% *CI*: 0.97–1.08; see online supplementary materials, Figure S3).

### Subgroup analysis and sensitivity analysis

Table 3 summarizes the results of the subgroup analysis according to several research characteristics. Subgroup analyses according to country/location (Asia/non-Asia) and parity-adjusted (yes/no) reduced the heterogeneity of the association between the consumption of fruit and GDM. We also assessed the period of dietary assessment (prepregnancy/first trimester/second trimester), the total number of participants (≥ 2000/<2000), the number of GDM patients (≥ 500/<500), pre-BMI (< 25/≥25 kg/m^2^), and adjustment factors such as parity (yes/no), family history of diabetes-adjusted (yes/no), physical activity (yes/no), smoking status (yes/no), and alcohol consumption (yes/no). Although statistically nonsignificant, some of the subgroups changed the effect size dramatically, such as the period of dietary assessment (prepregnancy), pre-BMI (≥ 25 kg/m^2^), and parity-adjusted (yes). As a result of the sensitivity analysis, the summary *RRs* for fruit, vegetable, and juice intake and GDM are as follows. Fruit intake: ranged from 0.92 (95% *CI*: 0.86–0.99) to 0.92 (95% *CI*: 0.85–0.98); all vegetable intake: ranged from 0.96 (95% *CI*: 0.90–1.03) to 0.98 (95% *CI*: 0.90–1.06); starchy vegetable intake: ranged from 0.78 (95% *CI*: 0.32–1.90) to 1.04 (95% *CI*: 0.97–1.13); fruit juice intake ranged from 0.97 (95% *CI*: 0.91–1.04) to 0.96 (95% *CI*: 0.90–1.03). Associations did not change considerably from the summary results (see online supplementary materials, Figure S4-S6).

### Publication bias

Based on the funnel plot, Begg’s tests (see online supplementary materials, Figure S6-S8), and Egger tests, no significant publication bias was found (fruit: *P*_Begg_= 0.216 and *P*_Egger_=0.191; all vegetable: *P*_Begg=_0.174 and *P*_Egger_=0.060; starchy vegetable: *P*_Begg=_0.602 and *P*_Egger_=0.622; fruit juice: *P*_Begg=_0.497 and *P*_Egger_=0.874) (Table 2).

## Discussion

The risk of GDM was inversely related to fruit consumption in this meta-analysis of 12 cohort studies, but no association was found between vegetables, fruit juices, and GDM. Moreover, a dose‒response analysis found that every 100 g of fruit per day reduced the risk of GDM by 3%. These findings offer support for the positive effects of moderate fruit intake on human health.

Studies have shown that fruit can reduce diabetes risk [41]. In regard to the relationship between diabetes and fruit consumption, there may be variations between different types of fruit. Rine et al. found that total fruit (apples, pears, blueberries, and grapes) and fruit combined with vegetables were related to a reduced risk of T2DM [42]. However, Wu-Qing Huang et al. found excessive intake (419 g/d) of tropical and citrus fruits, and fruits with a moderate or high glycaemic index (GI) increased the risk of GDM. The recommended intake of fruit during pregnancy is 200–400 g in China [32]. Studies have shown that most people currently fall short of the recommended daily intake of fruit [43, 44]. The average intake of fruit in this review was 220 g per day, which met the recommended intake. Thus, different fruit intakes could explain the inconsistent results of these studies. There are polyphenols, and antioxidant compounds in fruit, such as carotenoids and vitamins C and E. These compounds alleviate oxidative stress in cells that interfere with glucose uptake and prevent the development of abnormal glucose tolerance [45]. The fibre in fruits and vegetables can delay the absorption of carbohydrates from food and prevent a rapid rise in blood sugar [46]. The high fructose content of fruits is associated with impaired function of pancreatic β-cells and decreased insulin sensitivity [47–49]. The beneficial effects of fruits are determined not only by the effectiveness of specific micronutrients, but also by the combined action of many plant compounds. The high fructose content of fruits can be counteracted by the beneficial effects of fibre and other antioxidants [32]. Therefore, future studies can investigate the relationship between fruit intake and GDM risk through different types of fruit with different glycaemic indexs.

We found that vegetable intake and fruit juice were not significantly linked to the risk of GDM in this meta-analysis. Studies have reported that a diet pattern rich in vegetables and soy products can reduce the risk of GDM. For every one-quarter increase in vegetable pattern score, the risk of GDM was reduced by 3% [50]. Increased vegetable consumption may increase dietary fibre consumption and reduce fat intake, and is associated with a reduced risk of GDM [50, 51]. In this study, no association was found between the consumption of all vegetables (including starchy vegetables and other vegetables) or starchy vegetables and the risk of developing GDM. However, the study by Li et al. suggested that the total prepregnancy consumption of starchy vegetables, such as potatoes, was positively related to the risk of developing GDM [34]. Potatoes and other starchy vegetables are good quality carbohydrates and can be used as a substitute for a staple food [52]. The excessive intake of starchy vegetables can lead to a rapid rise in blood glucose after meals, which can damage pancreatic β-cells and increase the risk of GDM in the long term [53, 54]. Studies have shown that the risk of GDM increased by 8% for each additional serving of baked and boiled potatoes and French fries consumed [23]. The nutrients and biological effects of different types and cooking methods of vegetables play different roles in regulating blood glucose and insulin concentrations [55]. There is still a need for more research to assess the association with the risk of GDM based on the intake of some specific vegetables such as starchy and non-starchy vegetables, and leafy vegetables and legumes.

The fibre in the fruit juice is decreased and the sugar content are increased due to the commercial manufacturing process [56]. The study by Imamura et al. found that the consumption of artificially made sweet drinks and fruit juices was positively associated with the development of diabetes. [57]. Compared to pure fruit juice, fruit juice with added sugar increases the incidence of T2DM [58]. In addition, dietary guidelines in the United States indicate that consuming 75–224 ml of juice per day does not increase the risk of T2DM, cardiovascular and other diseases. In contrast, some short-term studies have found regular consumption of juice to be beneficial for cardiovascular health and blood pressure control [59]. Excess sugar in sweetened fruit juices can add to the body’s burden of regulating blood sugar, leading to a glycaemic load. The inconsistencies and bias between studies may be because most studies did not differentiate between pure fruit juice and sugar-sweetened juice [8]. In addition, compared to eating whole fruit, fruit juices can lead to overconsumption of fruit due to the lack of crude fibre, which has a satiating effect [60].

These results need to be interpreted with caution due to the lack of analysis of different types of fruits, vegetables, and juices. In the future, the relationship between fruits, vegetables, and juices needs to be further investigated by different subtypes.

### Strengths and limitations

To interpret the findings appropriately, it is important to acknowledge this study’s limitations. Owing to a limited number of included studies and low reported intakes of certain fruits, vegetables, and fruit juices, this study did not carry out a specific analysis of the impact of specific fruits, vegetables, and fruit juices on the risk of GDM. Furthermore, the description of the cooking method and fruit juice and whether sugar was added not specified. Although subgroup analyses were performed to adjust for confounding factors that may influence the occurrence of GDM, residual confounding factors such as the history of endocrine disease and mode of conception may still be present. The majority of studies used a food frequency scale (FFQ) and dietary records to estimate dietary intake. Dietary assessments based on FFQ may have recall bias and do not provide a detailed record of daily changes in food intake. The FFQ can reflect food intake over time. The dietary record provides a more objective picture of eating habits and detailed food preparation methods, mitigating interindividual differences but requiring a more detailed record and a higher level of compliance [61, 62]. Indeed, some health-related outcomes with GDM were poorly covered, and owing notably to rough estimates of the degree of fruit and vegetable processing, many associations were not reviewed systematically.

## Conclusion

Based on the results of this meta-analysis, fruit consumption appears to be associated with a reduced risk of GDM, whereas vegetable and fruit juice consumption was not associated with the incidence of GDM. According to the results of dose response analyses, increasing fruit consumption by 100 g per day reduced the risk of GDM by 3%. This suggests that people can reduce their risk of GDM by consuming moderate amounts of fresh fruit every day. Therefore, more high-quality, large-sample studies are needed to further investigate the relationship between different varieties of fruits, vegetables, and juices and the risk of GDM.

## Electronic supplementary material

Below is the link to the electronic supplementary material.

Supplementary Material 1

Supplementary Material 2

Supplementary Material 3

## Data Availability

Not applicable.
