# Supplementary material for: Fruit, vegetable, and fruit juice consumption and risk of gestational diabetes mellitus: a systematic review and meta-analysis: List of all authors
Source: Nutr J. 2023 May 20;22:27. doi: 10.1186/s12937-023-00855-8 (PMC10199474; doi:10.1186/s12937-023-00855-8)
Supplement: Supplementary file 1 — Supplementary Material 1 [file 12937_2023_855_MOESM1_ESM.pdf]

SPRINGER NATURE
Author Services

Editing Certificate

This document certifies that the manuscript

Fruit, vegetable, and fruit juice consumption and risk of gestational diabetes mellitus: a systematic review and meta-analysis

prepared by the authors

Yan-Ping Liao, Qing-Xiang Zheng, Xiu-Min Jiang *, Xiao-Qian Chen, Xiao-Xia Gao , Yu-Qing Pan

was edited for proper English language, grammar, punctuation, spelling, and overall style by one or more of the highly qualified native English speaking editors at SNAS.

This certificate was issued on **March 14, 2023** and may be verified on the SNAS website using the verification code **50DB-59F3-B3D1-2B1D-A8CC** .

Neither the research content nor the authors' intentions were altered in any way during the editing process. Documents receiving this certification should be English-ready for publication; however, the author has the ability to accept or reject our suggestions and changes. To verify the final SNAS edited version, please visit our verification page at secure.authorservices.springernature.com/certificate/verify.
If you have any questions or concerns about this edited document, please contact SNAS at support@as.springernature.com.

SNAS provides a range of editing, translation, and manuscript services for researchers and publishers around the world.
For more information about our company, services, and partner discounts, please visit authorservices.springernature.com.
