# Supplementary material for: Fruit, vegetable, and fruit juice consumption and risk of gestational diabetes mellitus: a systematic review and meta-analysis: List of all authors
Source: Nutr J. 2023 May 20;22:27. doi: 10.1186/s12937-023-00855-8 (PMC10199474; doi:10.1186/s12937-023-00855-8)
Supplement: Supplementary file 2 — Supplementary Material 2 [file 12937_2023_855_MOESM2_ESM.docx]

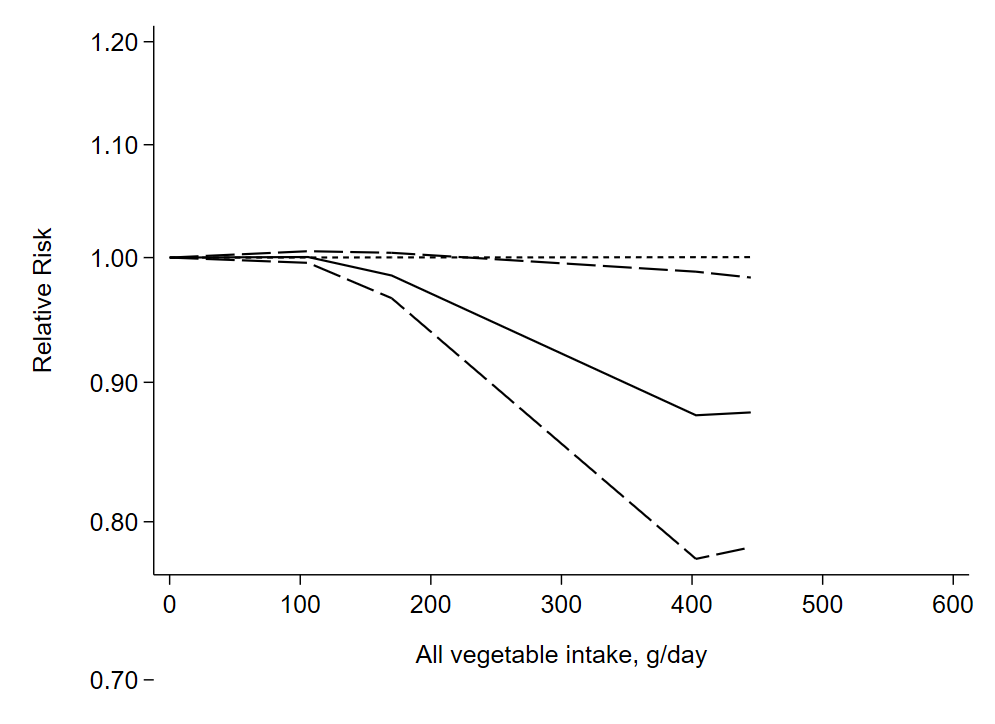


**Figure S1**. Dose–response analyses of all vegetable intake and risk of GDM.


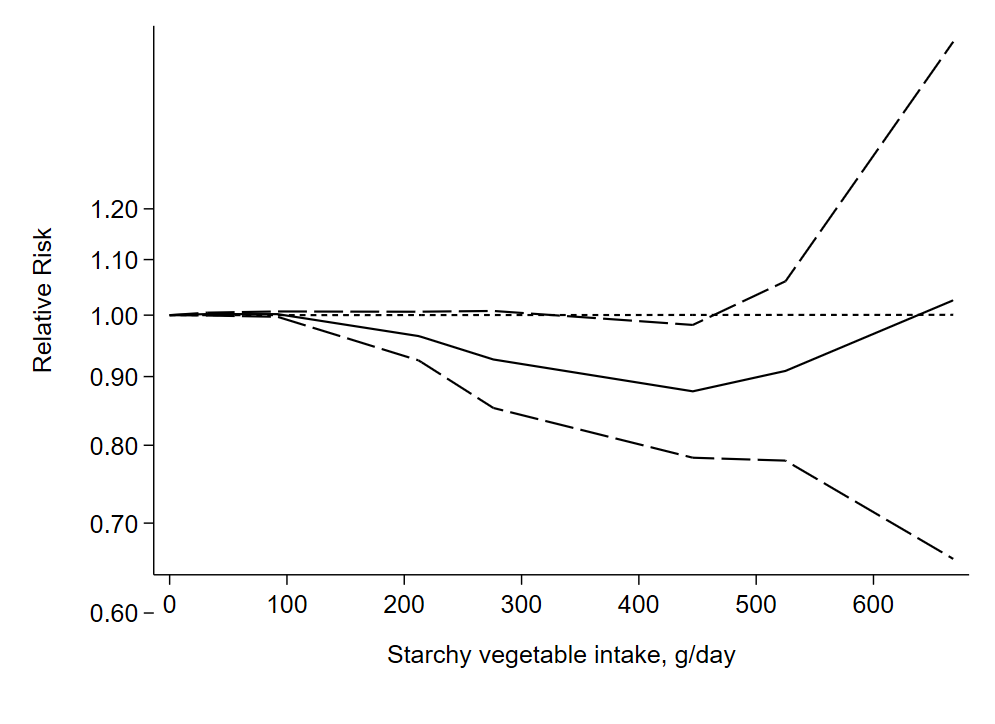


**Figure S2**.Dose–response analyses of starchy vegetable intake and risk of GDM.


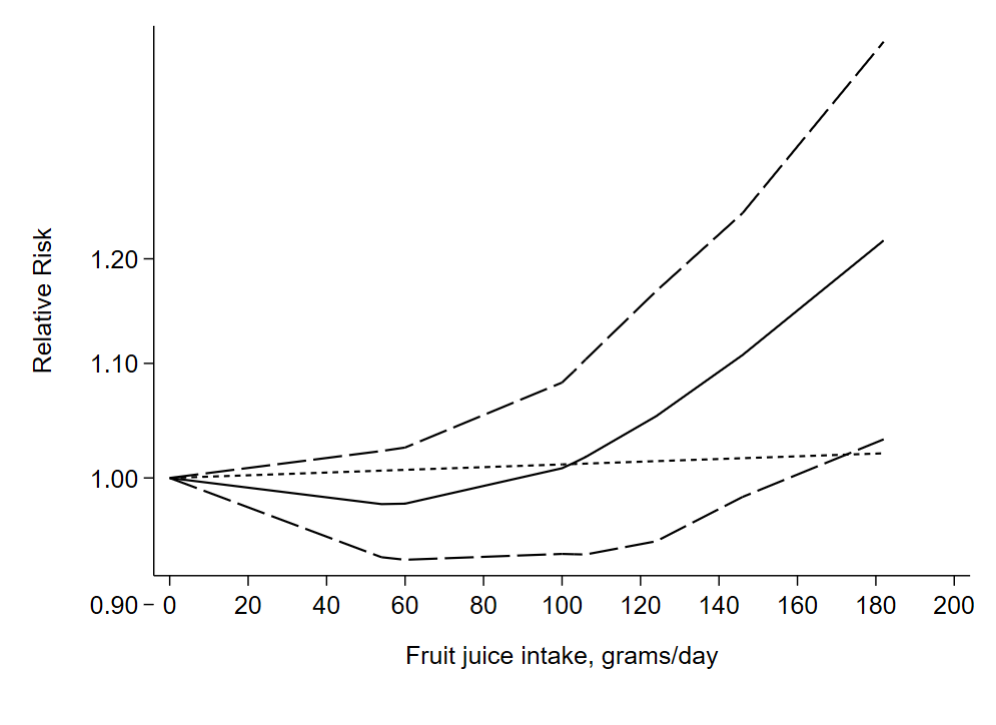


**Figure S3.** Dose–response analyses of fruit juice intake and risk of GDM.


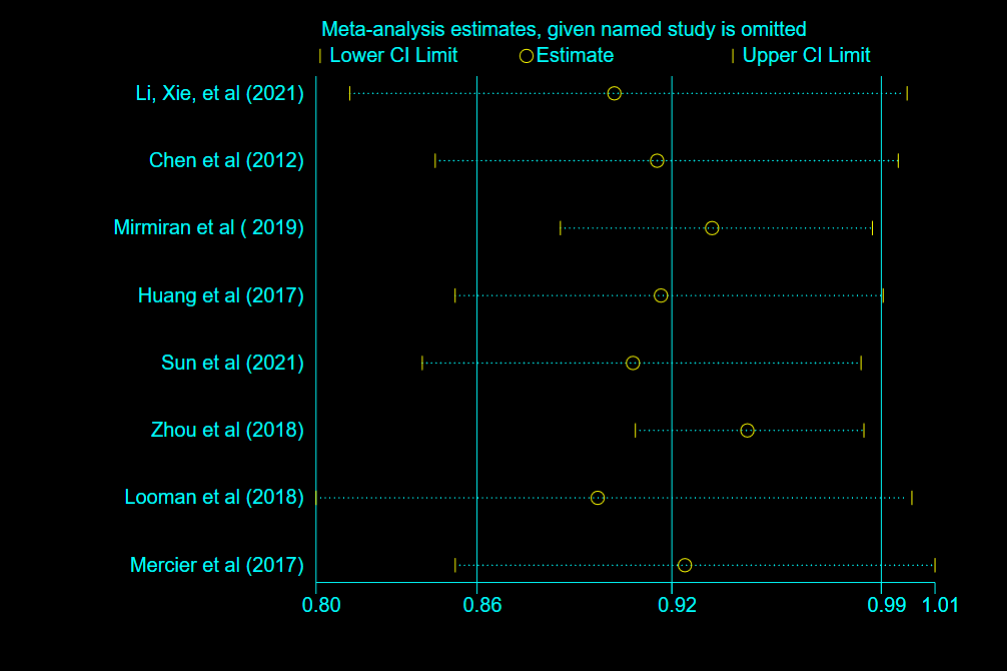


**Figure S4**. Sensitivity analysis of fruit intake and the GDM.


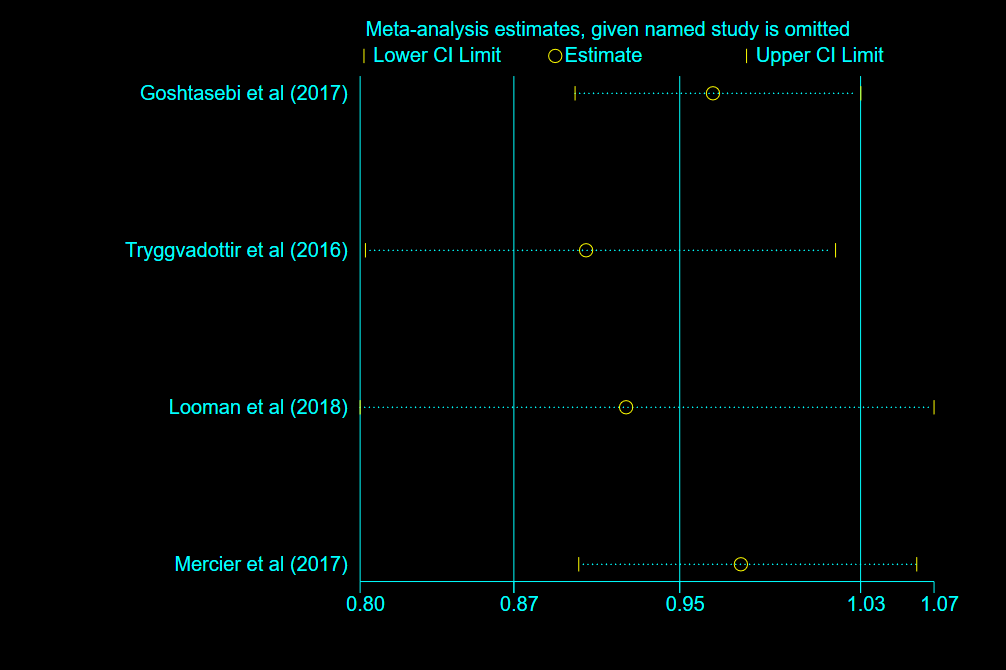


**Figure S5**. Sensitivity analysis of all vegetable intake and the GDM


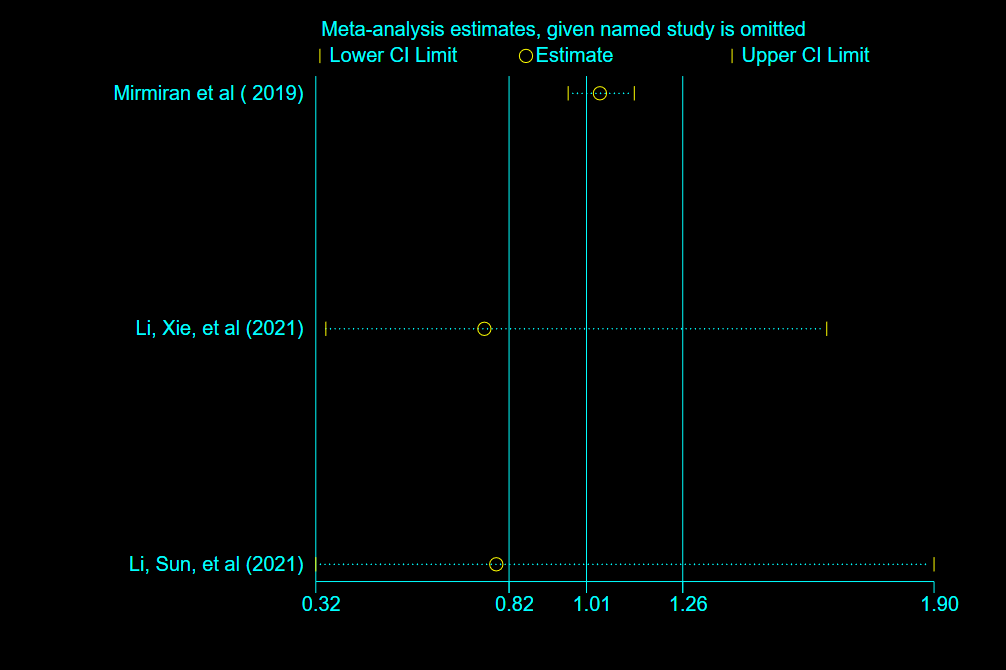


**Figure S6**. Sensitivity analysis of starchy vegetable intake and the GDM


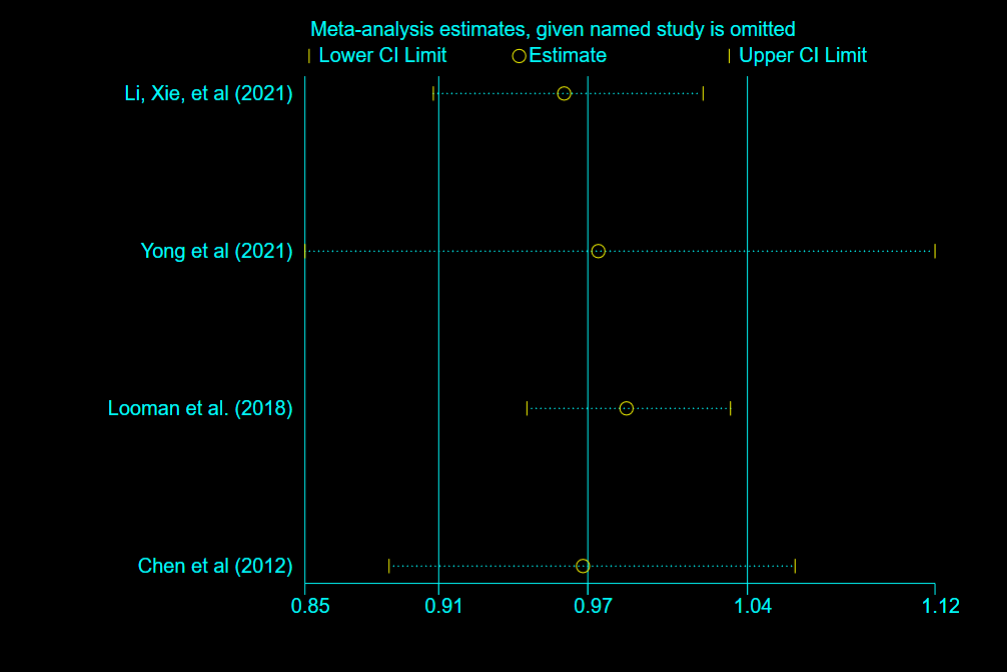


**Figure S7**. Sensitivity analysis of fruit juice intake and the GDM


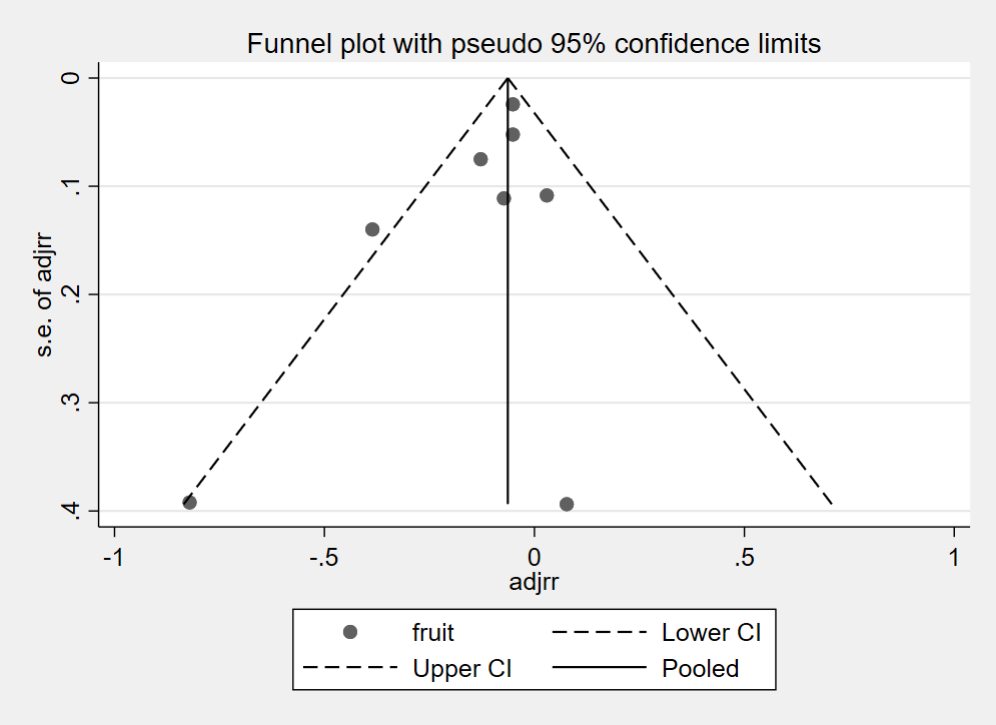


**Figure S8.** Funnel plot of fruit intake and GDM risk


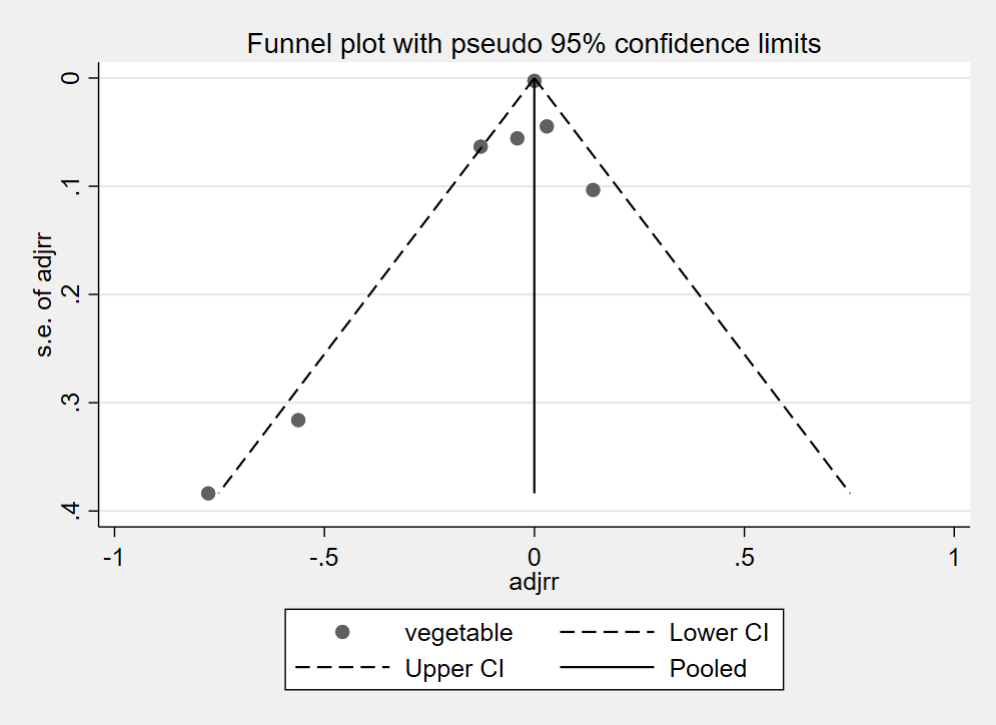


**Figure S9.** Funnel plot of vegetable intake and GDM risk


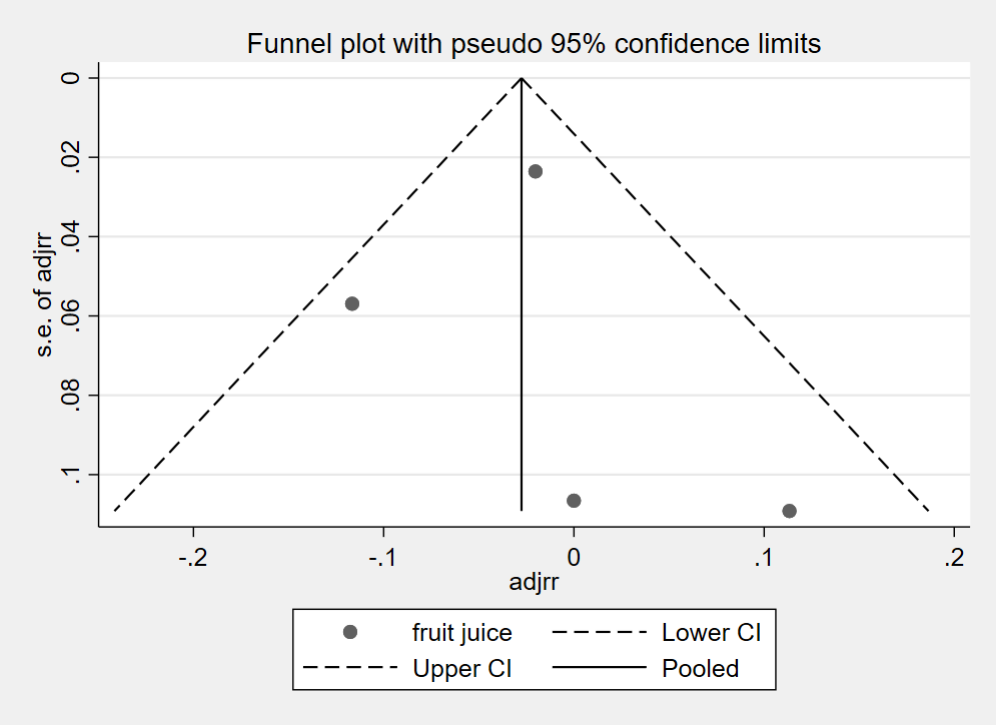


**Figure S10.** Funnel plot of fruit juice intake and GDM risk
