# Supplementary material for: Fruit, vegetable, and fruit juice consumption and risk of gestational diabetes mellitus: a systematic review and meta-analysis: List of all authors
Source: Nutr J. 2023 May 20;22:27. doi: 10.1186/s12937-023-00855-8 (PMC10199474; doi:10.1186/s12937-023-00855-8)
Supplement: Supplementary file 3 — Supplementary Material 3 [file 12937_2023_855_MOESM3_ESM.docx]

| **Section and Topic** | **Item #** | **Checklist item** | **Location where item is reported on page** |
| --- | --- | --- | --- |
| **TITLE** | | |  |
| Title | 1 | Identify the report as a systematic review. | 1 |
| **ABSTRACT** | | |  |
| Abstract | 2 | See the PRISMA 2020 for Abstracts checklist. | 1 |
| **INTRODUCTION** | | |  |
| Rationale | 3 | Describe the rationale for the review in the context of existing knowledge. | 1-2 |
| Objectives | 4 | Provide an explicit statement of the objective(s) or question(s) the review addresses. | 1-2 |
| **METHODS** | | |  |
| Eligibility criteria | 5 | Specify the inclusion and exclusion criteria for the review and how studies were grouped for the syntheses. | 3 |
| Information sources | 6 | Specify all databases, registers, websites, organisations, reference lists and other sources searched or consulted to identify studies. Specify the date when each source was last searched or consulted. | 3 |
| Search strategy | 7 | Present the full search strategies for all databases, registers and websites, including any filters and limits used. | 2 |
| Selection process | 8 | Specify the methods used to decide whether a study met the inclusion criteria of the review, including how many reviewers screened each record and each report retrieved, whether they worked independently, and if applicable, details of automation tools used in the process. | 3 |
| Data collection process | 9 | Specify the methods used to collect data from reports, including how many reviewers collected data from each report, whether they worked independently, any processes for obtaining or confirming data from study investigators, and if applicable, details of automation tools used in the process. | 3 |
| Data items | 10a | List and define all outcomes for which data were sought. Specify whether all results that were compatible with each outcome domain in each study were sought (e.g. for all measures, time points, analyses), and if not, the methods used to decide which results to collect. | Table1 |
|  | 10b | List and define all other variables for which data were sought (e.g. participant and intervention characteristics, funding sources). Describe any assumptions made about any missing or unclear information. | Table1 |
| Study risk of bias assessment | 11 | Specify the methods used to assess risk of bias in the included studies, including details of the tool(s) used, how many reviewers assessed each study and whether they worked independently, and if applicable, details of automation tools used in the process. | 3-4 |
| Effect measures | 12 | Specify for each outcome the effect measure(s) (e.g. risk ratio, mean difference) used in the synthesis or presentation of results. | 4 |
| Synthesis methods | 13a | Describe the processes used to decide which studies were eligible for each synthesis (e.g. tabulating the study intervention characteristics and comparing against the planned groups for each synthesis (item #5)). | 4 |
|  | 13b | Describe any methods required to prepare the data for presentation or synthesis, such as handling of missing summary statistics, or data conversions. | 4 |
|  | 13c | Describe any methods used to tabulate or visually display results of individual studies and syntheses. | Figure 1 and Table 2 |
|  | 13d | Describe any methods used to synthesize results and provide a rationale for the choice(s). If meta-analysis was performed, describe the model(s), method(s) to identify the presence and extent of statistical heterogeneity, and software package(s) used. | 4 |
|  | 13e | Describe any methods used to explore possible causes of heterogeneity among study results (e.g. subgroup analysis, meta-regression). | 4 |
|  | 13f | Describe any sensitivity analyses conducted to assess robustness of the synthesized results. | 4 |
| Reporting bias assessment | 14 | Describe any methods used to assess risk of bias due to missing results in a synthesis (arising from reporting biases). | 4 |
| Certainty assessment | 15 | Describe any methods used to assess certainty (or confidence) in the body of evidence for an outcome. | no |
| **RESULTS** | | |  |
| Study selection | 16a | Describe the results of the search and selection process, from the number of records identified in the search to the number of studies included in the review, ideally using a flow diagram. | 4 and Figure 1 |
|  | 16b | Cite studies that might appear to meet the inclusion criteria, but which were excluded, and explain why they were excluded. | 4 and Figure 1 |
| Study characteristics | 17 | Cite each included study and present its characteristics. | Table 1 |
| Risk of bias in studies | 18 | Present assessments of risk of bias for each included study. | Table 1 |
| Results of individual studies | 19 | For all outcomes, present, for each study: (a) summary statistics for each group (where appropriate) and (b) an effect estimate and its precision (e.g. confidence/credible interval), ideally using structured tables or plots. | Figure 2 and Table 2 |
| Results of syntheses | 20a | For each synthesis, briefly summarise the characteristics and risk of bias among contributing studies. | 4 and Table2 |
|  | 20b | Present results of all statistical syntheses conducted. If meta-analysis was done, present for each the summary estimate and its precision (e.g. confidence/credible interval) and measures of statistical heterogeneity. If comparing groups, describe the direction of the effect. | Figure 2 and Table 2 |
|  | 20c | Present results of all investigations of possible causes of heterogeneity among study results. | 11-12 |
|  | 20d | Present results of all sensitivity analyses conducted to assess the robustness of the synthesized results. | 11-12 |
| Reporting biases | 21 | Present assessments of risk of bias due to missing results (arising from reporting biases) for each synthesis assessed. | 12 and Table 3 |
| Certainty of evidence | 22 | Present assessments of certainty (or confidence) in the body of evidence for each outcome assessed. | 12 |
| **DISCUSSION** | | |  |
| Discussion | 23a | Provide a general interpretation of the results in the context of other evidence. | 12 |
|  | 23b | Discuss any limitations of the evidence included in the review. | 12-14 |
|  | 23c | Discuss any limitations of the review processes used. | 12-14 |
|  | 23d | Discuss implications of the results for practice, policy, and future research. | 12-14 |
| **OTHER INFORMATION** | | |  |
| Registration and protocol | 24a | Provide registration information for the review, including register name and registration number, or state that the review was not registered. | no |
|  | 24b | Indicate where the review protocol can be accessed, or state that a protocol was not prepared. | no |
|  | 24c | Describe and explain any amendments to information provided at registration or in the protocol. | no |
| Support | 25 | Describe sources of financial or non-financial support for the review, and the role of the funders or sponsors in the review. | 15 |
| Competing interests | 26 | Declare any competing interests of review authors. | 15 |
| Availability of data, code and other materials | 27 | Report which of the following are publicly available and where they can be found: template data collection forms; data extracted from included studies; data used for all analyses; analytic code; any other materials used in the review. | 15 |

*From:*  Page MJ, McKenzie JE, Bossuyt PM, Boutron I, Hoffmann TC, Mulrow CD, et al. The PRISMA 2020 statement: an updated guideline for reporting systematic reviews. BMJ 2021;372:n71. doi: 10.1136/bmj.n71

For more information, visit: <http://www.prisma-statement.org/>

**Table S2 Search Strategy**

**Mesh terms and free words**

**（1）English:**

| Concept | | Keywords | |
| --- | --- | --- | --- |
| Participants | Mesh terms | pregnancy |  |
|  | Free words | pregnan* | gestation* |
| Intervention | Mesh terms | fruit | vegetables |
|  |  | fruit and vegetable juices |  |
|  | Free words | product, vegetable | vegetable |
|  |  | fruits |  |
|  |  | vegetable juices or juice | vegetable or juices |
|  |  | vegetable juices | juice, vegetable |
|  |  | juices, vegetable | vegetable juice |
|  |  | fruit juices | juices, fruit |
| Outcomes | Mesh terms | diabetes, gestational |  |
|  | Free words | diabetes, pregnancy-induced | diabetes, pregnancy induced |
|  |  | pregnancy-induced diabetes | gestational diabetes |
|  |  | diabetes mellitus, gestational | gestational diabetes mellitus |
| Study design | Mesh terms | cohort studies |  |
|  |  | cohort study | studies, cohort |
|  |  | concurrent studies | closed cohort studies |

**（2）Chinese:**

| Concept | | Keywords | |
| --- | --- | --- | --- |
| Participants | Mesh terms | 妊娠 |  |
|  | Free words | 生产 | 怀孕 |
|  |  | 孕妇 | 产妇 |
|  |  | 分娩 |  |
| Intervention | Mesh terms | 水果 | 蔬菜 |
|  |  | 果汁 |  |
|  | Free words | 蔬菜水果 | 水果蔬菜 |
|  |  | 果蔬汁 |  |
| Outcomes | Mesh terms | 妊娠期糖尿病 |  |
| Study design | Mesh terms | 队列研究 |  |

**一、PubMed**

#1 (((pregnancy [MeSH Terms])) OR (pregnan*[Title/Abstract])) OR (gestation* [Title/Abstract])

#2 (((((((((((((fruit [MeSH Terms]) OR (vegetables [MeSH Terms])) OR (fruit and vegetable juices [MeSH Terms])) OR (vegetable [Title/Abstract])) OR (fruits [Title/Abstract])) OR (vegetable juices[Title/Abstract] OR juice [Title/Abstract])) OR juices [Title/Abstract])) OR (vegetable juices [Title/Abstract])) OR (juice, vegetable [Title/Abstract])) OR (juices, vegetable [Title/Abstract])) OR (vegetable juice [Title/Abstract])) OR (fruit juices [Title/Abstract])) OR (juices, fruit [Title/Abstract])

#3 ((((((diabetes, gestational [MeSH Terms]) OR (diabetes, pregnancy-induced [Title/Abstract])) OR (diabetes, pregnancy induced [Title/Abstract])) OR (pregnancy-induced diabetes [Title/Abstract])) OR (gestational diabetes [Title/Abstract])) OR (diabetes mellitus, gestational [Title/Abstract])) OR (gestational diabetes mellitus [Title/Abstract])

#4 (((((cohort studies [MeSH Terms])) OR (cohort study [Title/Abstract])) OR (studies, cohort [Title/Abstract])) OR (concurrent studies [Title/Abstract])) OR (closed cohort studies [Title/Abstract])

#5 #1 and #2 and #3 and #4

**二、the Cochrane Library**

#1 (pregnancy or pregnan*or gestation*): ti,ab,kw

#2 (fruit or vegetables or fruit and vegetable juices or vegetable or fruits or vegetable juices or juice or vegetable or juices or vegetable juices or juice, vegetable or juices, vegetable or vegetable juice or fruit juices or juices, fruit): ti,ab,kw

#3 (diabetes, gestational or diabetes, pregnancy-induced or diabetes, pregnancy induced or pregnancy-induced diabetes or gestational diabetes or diabetes mellitus, gestational or gestational diabetes mellitus): ti,ab,kw

#4 (cohort studies or cohort study or studies, cohort or concurrent studies or closed cohort studies): ti,ab,kw

#5 #1 and #2 and #3 and #4

**三、Web of science**

#1 TS= (pregnancy or pregnan*or gestation*)

#2 TS= (fruit or vegetables or fruit and vegetable juices or vegetable or fruits or vegetable juices or juice or vegetable or juices or vegetable juices or juice, vegetable or juices, vegetable or vegetable juice or fruit juices or juices, fruit)

#3 TS= (diabetes, gestational or diabetes, pregnancy-induced or diabetes, pregnancy induced or pregnancy-induced diabetes or gestational diabetes or diabetes mellitus, gestational or gestational diabetes mellitus)

#4 TS= (cohort studies or cohort study or studies, cohort or concurrent studies or closed cohort studies)

#5 #1 and #2 and #3 and #4

**四、Embase**

#1 pregnancy:ab,ti or 'pregnan*or gestation*':ab,ti

#2 vegetables:ab,ti or fruit:ab,ti or 'vegetable juices':ab,ti or 'vegetable':ab,ti or fruits:ab,ti or juice:ab,ti or vegetable:ab,ti or juices:ab,ti or 'vegetable juices':ab,ti or 'juice, vegetable':ab,ti or 'juices, vegetable':ab,ti or 'vegetable juice':ab,ti or 'fruit juices':ab,ti or 'juices, fruit':ab,ti

#3 'diabetes, gestational':ab,ti or 'diabetes, pregnancy-induced':ab,ti or 'diabetes, pregnancy induced':ab,ti or 'pregnancy-induced diabetes':ab,ti or 'gestational diabetes':ab,ti or 'diabetes mellitus, gestational':ab,ti or 'gestational diabetes mellitus':ab,ti

#4 'cohort studies’: ab,ti or 'cohort study':ab,ti or 'studies, cohort':ab,ti or 'concurrent studies':ab,ti or 'closed cohort studies':ab,ti

#5 #1 and #2 and #3 and #4

**五、Ovid**

1 (pregnancy or pregnan*or gestation*).mp. [mp=title, abstract, original title, name of substance word, subject heading word, floating sub-heading word, keyword heading word, organism supplementary concept word, protocol supplementary concept word, rare disease supplementary concept word, unique identifier, synonyms]

2 (fruit or vegetables or fruit and vegetable juices or vegetable or fruits or vegetable juices or juice or vegetable or juices or vegetable juices or juice, vegetable or juices, vegetable or vegetable juice or fruit juices or juices, fruit).mp. [mp=title, abstract, original title, name of substance word, subject heading word, floating sub-heading word, keyword heading word, organism supplementary concept word, protocol supplementary concept word, rare disease supplementary concept word, unique identifier, synonyms]

3 (diabetes, gestational or diabetes, pregnancy-induced or diabetes, pregnancy induced or pregnancy-induced diabetes or gestational diabetes or diabetes mellitus, gestational or gestational diabetes mellitus).mp. [mp=title, abstract, original title, name of substance word, subject heading word, floating sub-heading word, keyword heading word, organism supplementary concept word, protocol supplementary concept word, rare disease supplementary concept word, unique identifier, synonyms]

4 (cohort studies or cohort study or studies, cohort or concurrent studies or closed cohort studies).mp. [mp=title, abstract, original title, name of substance word, subject heading word, floating sub-heading word, keyword heading word, organism supplementary concept word, protocol supplementary concept word, rare disease supplementary concept word, unique identifier, synonyms]

5 1 and 2 and 3 and 4

**六、EBSCO**

S1 AB pregnancy or pregnan*or gestation*

S2 AB fruit or vegetables or fruit and vegetable juices or vegetable or fruits or vegetable juices or juice or vegetable or juices or vegetable juices or juice, vegetable or juices, vegetable or vegetable juice or fruit juices or juices, fruit

S3 AB diabetes, gestational or diabetes, pregnancy-induced or diabetes, pregnancy induced or pregnancy-induced diabetes or gestational diabetes or diabetes mellitus, gestational or gestational diabetes mellitus

S4 cohort studies or cohort study or studies, cohort or concurrent studies or closed cohort studies

S5 S1 and S2 and S3 and S4

七、Scopus

TITLE-ABS (gestational AND diabetes) AND TITLE-ABS (vegetables) AND TITLE-ABS (fruits) AND TITLE-ABS (cohort)

八、知网

SU= '妊娠' + '生产' + '怀孕' + '孕妇' + '产妇' + '分娩' and SU='蔬菜'+'水果'+'果汁'+'蔬菜水果'+'水果蔬菜' +'果蔬汁' and SU='妊娠期糖尿病' and SU='队列研究'

九、万方

题名或关键词:( 妊娠 or 生产 or怀孕 or 孕妇 or 产妇 or 分娩 ) and 题名或关键词:(蔬菜or水果or果汁or蔬菜水果or水果蔬菜or果蔬汁) and 题名或关键词:(妊娠期糖尿病) and 题名或关键词:(队列研究)

十、维普

(M=妊娠or M=生产 or M=怀孕 or M=孕妇 or M=产妇 or M=分娩) and ( M=蔬菜or M=水果 or M=果汁or M=蔬菜水果 or M=水果蔬菜 or M=果蔬汁）and ( M=妊娠期糖尿病）and ( M=队列研究）
